# In-depth analysis of pre- and postoperative functional outcome parameters in patients receiving laryngotracheal surgery

**DOI:** 10.1093/ejcts/ezae171

**Published:** 2024-04-19

**Authors:** Matthias Evermann, Imme Roesner, Doris-Maria Denk-Linnert, Walter Klepetko, Thomas Schweiger, Konrad Hoetzenecker

**Affiliations:** Department of Thoracic Surgery, Medical University of Vienna, Vienna, Austria; Division of Phoniatrics and Logopedics, Department of Otorhinolaryngology, Medical University of Vienna, Vienna, Austria; Division of Phoniatrics and Logopedics, Department of Otorhinolaryngology, Medical University of Vienna, Vienna, Austria; Department of Thoracic Surgery, Medical University of Vienna, Vienna, Austria; Department of Thoracic Surgery, Medical University of Vienna, Vienna, Austria; Department of Thoracic Surgery, Medical University of Vienna, Vienna, Austria

**Keywords:** Laryngotracheal surgery, Laryngotracheal reconstruction, Subglottic stenosis, Cricotracheal resection, Laryngotracheal stenosis

## Abstract

**OBJECTIVES:**

Surgical treatment for airway stenosis necessitates personalized techniques based on the stenosis location and length, leading to favourable surgical outcomes. However, there is limited literature on functional outcomes following laryngotracheal surgery with an adequate number of patients.

**Methods:**

We conducted a retrospective analysis of patients who underwent laryngotracheal surgery at the Department of Thoracic Surgery, Medical University of Vienna, from January 2017 to June 2021. The study included standardized functional assessments before and after surgery, encompassing spirometry, voice measurements, swallowing evaluation and subjective patient perception.

**RESULTS:**

The study comprised 45 patients with an average age of 51.9 ± 15.9 years, of whom 89% were female, with idiopathic being the most common aetiology (67%). Procedures included standard cricotracheal resection in 11%, cricotracheal resection with dorsal mucosal flap in 49%, cricotracheal resection with dorsal mucosal flap and lateral cricoplasty in 24% and single-stage laryngotracheal reconstruction in 16%. There were no in-hospital mortalities or restenosis cases during the mean follow-up period of 20.8 ± 13.2 months. Swallowing function remained intact in all patients. Voice evaluations showed a decrease in fundamental vocal pitch [203 (81–290) Hz vs 150 (73–364) Hz, *P* < 0.001] and dynamic voice range (23.5 ± 5.8 semitones vs 17.8 ± 6.7 semitones, *P* < 0.001). However, no differences in voice volume were observed (60.0 ± 4.1 dB vs 60.2 ± 4.8 dB, *P* = 0.788). The overall predicted voice profile changed from R0B0H0 to R1B0H1.

**CONCLUSIONS:**

Laryngotracheal surgery proves effective in fully restoring breathing capacity while preserving vocal function. Even in cases of high-grade and complex airway stenosis necessitating laryngotracheal reconstruction, favourable functional outcomes can be achieved.

## Graphical Abstract

**Figure:**
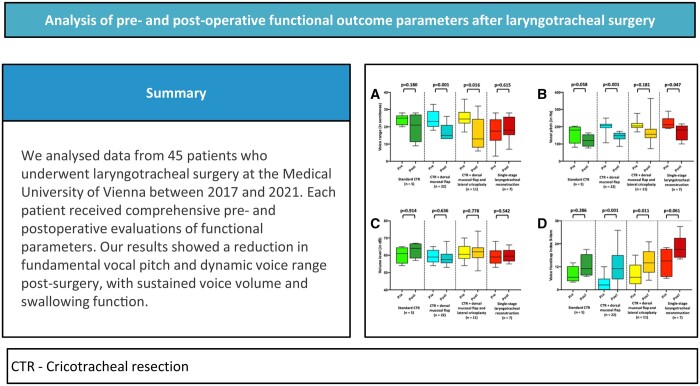

## INTRODUCTION

Surgical treatment of laryngotracheal stenoses is considered demanding due to the functional peculiarities of the laryngotracheal junction and the low overall number of procedures performed even in specialized centres. The major aim of the surgical treatment is the complete removal of the cicatricial tissue and subsequent anastomosis of healthy mucosa. Proper surgery leads to unrivaled low numbers of restenosis compared to endoscopic treatment alternatives, such as laser resection and balloon dilatation [1].

On the other hand, the potential negative impact of surgery on the postoperative quality of voice often leads to reservations regarding surgical treatment options. This is especially relevant for surgical resections involving the subglottic space. The partial removal of the cricoid arch affects the functioning of the larynx and results in an altered postoperative voice [2, 3]. Recently, a number of modifications to the standard cricotracheal resection (CTR) have been described, but the functional impact of these modification is still poorly understood [4–6]. Furthermore, a pre-existing impairment of the voice function, which can frequently be found in patients with stenoses extending towards the glottis, has to be taken into account. However, a detailed pre- and postoperative diagnostic work-up of the laryngeal functioning is only rarely reported [7, 8]. Interpreting the extent of the postoperative loss of voice function is therefore hardly possible.

In this study, we aimed to summarize the pre- and postoperative functional characteristics of patients receiving laryngotracheal surgery. Additionally, the impact of various surgical techniques on the laryngeal functioning was described.

## MATERIALS AND METHODS

### Study population

All patients who underwent laryngotracheal surgery at the Department of Thoracic Surgery, Medical University of Vienna, between January 2017 and June 2021 were retrospectively included in this single-centre study. Patients who underwent resections confined to the trachea, paediatric patients and patients with malignant aetiologies were excluded. We also excluded patients who were unable to perform a functional measurement preoperatively. Medical records were analysed to define patient characteristics and clinical variables, medical history, functional as well as endoscopic measurements, surgical procedures and long-term follow-up. The study was approved by the Ethics Committee of the Medical University of Vienna (1639/2023).

### Surgical management

**Figure 1:**
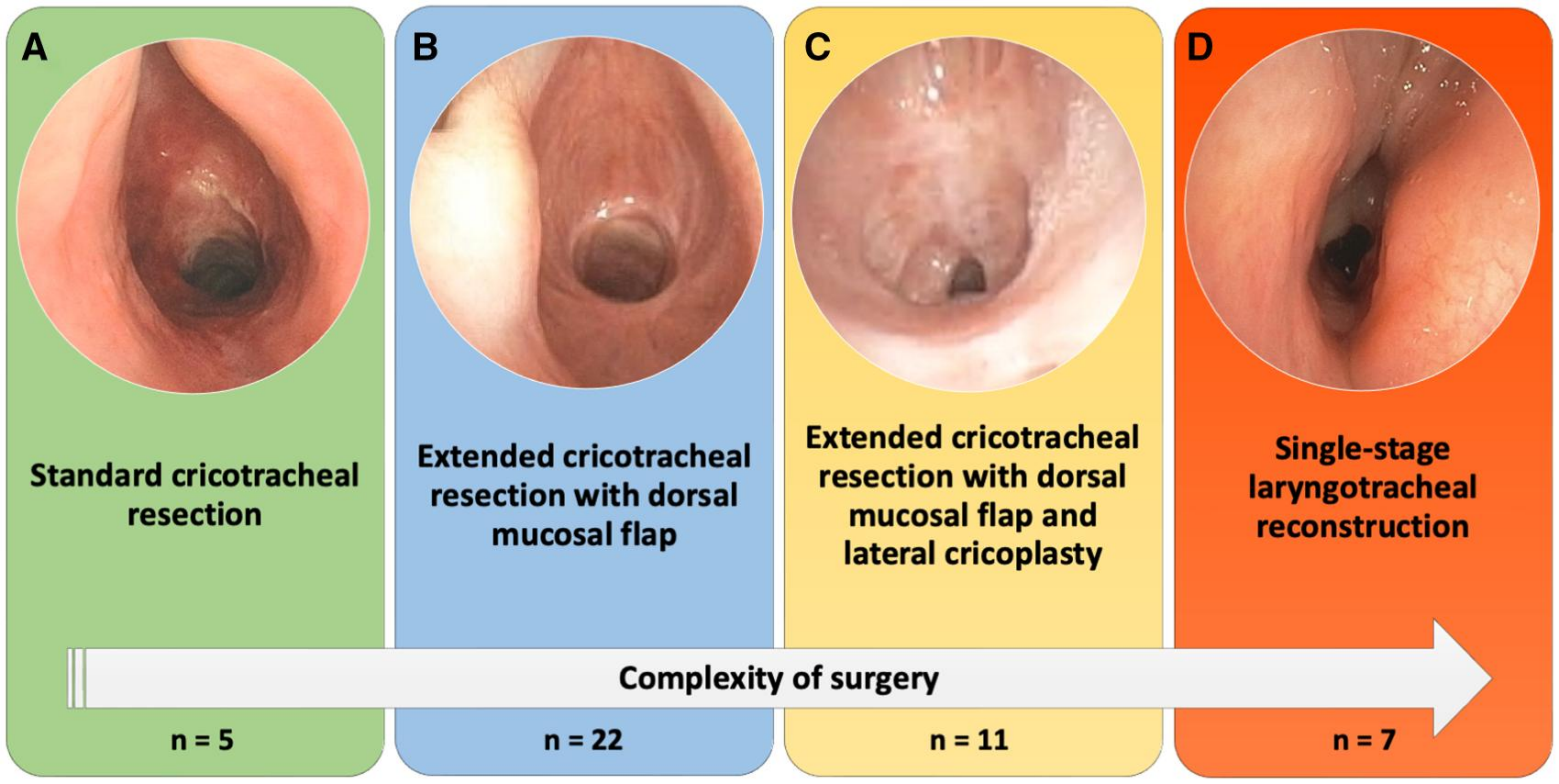
Pre-operative bronchoscopic images of a patient receiving (**A**) standard cricotracheal resection, (**B**) extended cricotracheal resection with dorsal mucosal flap, (**C**) extended cricotracheal resection with dorsal mucosal flap and lateral cricoplasty and (**D**) single-stage laryngotracheal reconstruction.

Cervicotomy represented the standard approach for surgical treatment of laryngotracheal stenosis. After induction of anaesthesia, a laryngeal mask was placed [9]. A cervical incision and midline dissection to the cricoid level were performed. The trachea was transected below the stenosis, and ventilation was started switched to cross-table ventilation with intermitted apnoea. The various techniques of laryngotracheal surgery have now been described widely [4]. Depending on the extent and location of the stenosis, a standard CTR [10], a CTR with dorsal mucosal flap [11], a CTR with dorsal mucosal flap and lateral cricoplasty [12] or a single-stage laryngotracheal reconstruction (SSLTR) [13] was performed (Fig. 1).

#### Standard CTR

In standard CTR (Fig. 1A), the anterior cricoid arch was removed. In this procedure, an incision was performed through the cricothyroid membrane and the stenotic segment was removed with the cricoid arch. Subsequently, a thyro-tracheal end-to-end anastomosis was performed.

#### CTR with dorsal mucosectomy

In case of extensive scarring on the posterior cricoid plate (Fig. 1B), a CTR with dorsal mucosectomy and was performed. In this procedure, the scar tissue of the dorsal subglottis was 1st removed and then covered with a mucosa flap, which was created from the pars membranacea of the distal trachea.

#### CTR with dorsal mucosectomy and lateral cricoplasty

A modified CTR with dorsal mucosectomy and a lateral cricoplasty has been used for severe side-to-side stenosis of the subglottis (Fig. 1C). In this technique, 1st described by Liberman and Mathisen [12], the submucosa and part of the lateral cricoid arch was reduced on both sides by resection auf the mucosa. Then the remaining mucosa was fixed to the outside in order to obtain an enlargement of the lumen.

#### Single-stage laryngotracheal reconstruction

Especially in high-grade glotto-subglottic stenosis (Fig. 1D), SSLTR has been performed. In this technique, which has been modified at our centre [13], the larynx was 1st split and a cartilage graft was inserted posteriorly to widen the lumen. Afterwards, the cartilage was covered with a dorsal mucosal flap, whereby no internal stenting was required postoperatively [14].

### Functional evaluation

All patients included in this study underwent an extensive functional evaluation before surgery and 3 months postoperatively (Supplementary Material, Fig. S1). A voice evaluation was conducted by an experienced phoniatrician including a Roughness–Breathiness–Hoarseness scoring for roughness, breathiness and hoarseness of the voice from 0 (normal) to 3 (severe impairment), a 9-item Voice Handicap Index (VHI) and phonation time. Vocal pitch (in Hz), voice range (in semitones) and fundamental volume level (in dB) were measured by DiVAS software from XION Medical (Berlin, Germany). Fibreoptic endoscopic evaluation of swallowing for liquid, semi-solid and solid consistencies is performed. This included an assessment of the vocal cord movement, level of phonation and glottic closure. In addition, a patient self-rating of the severity of dysphagia from 1 (no impairment) to 7 (severe dysphagia) is performed. Spirometry was also performed by each patient before and 3 months after surgery to quantify airway patency.

### Statistical analysis

Statistical analysis was performed using SPSS 21 (SPSS Inc., Chicago, USA) and GraphPad Prism 6 (GraphPad Software Inc., California, USA). Chi-square test and Fisher’s exact test were used to compare binominal variables. Student’s *t*-test was used to compare variables of 2 independent normally distributed groups, otherwise the Mann–Whitney *U*-test was used. In order to compare more than 2 variables, the Kruskal–Wallis test was chosen. Anderson–Darling test was used to assess normal assumption. Normally distributed variables were reported with mean ± standard deviation, otherwise they were presented with median and range. Categorical variables were presented as values and percentages. All tests were two-sided unless otherwise noted. *P*-values <0.05 were considered as statistically significant.

## RESULTS

### Patient characteristics

A total of 45 patients with a mean age of 51.9 ± 15.9 years were included in the study. Forty (89%) patients were female. The most common aetiology was idiopathic (67%) stenosis, followed by acquired (18%) and autoimmune causes (15%). Twenty-three (51%) of the patients had been pretreated with a median number of 2 endoscopic procedures (range 1–9) before they were referred for a surgical repair. The vast majority of stenoses were classified as high-grade Myer–Cotton [15] grade III (89%), and the remainder as grade II (11%). Median length of stenosis was 20 mm (range 5–45) with a median total length of trachea of 120 mm (range 105–135). On average, the stenoses extended up to 3 mm to the vocal fold level (range 3–30). Three (7%) patients had permanent tracheostomy before surgery (Table 1).

**Table 1:** Patient characteristics and clinical variables in patients with laryngotracheal stenoses

Variable	Total (*n* = 45)	Standard CTR (*n* = 5)	CTR with dorsal flap (*n* = 22)	CTR with lateral cricoplasty (*n* = 11)	SSLTR (*n* = 7)	*P*-value
	*n* (%)	*n* (%)	*n* (%)	*n* (%)	*n* (%)	
Sex						
Male	5 (11)	2 (40)	2 (9)	1 (9)	0 (0)	0.162
Female	40 (89)	3 (60)	20 (91)	10 (91)	7 (100)	
Age at surgery (years)	51.9 ± 15.9	53.9 ± 13.4	51.3 ± 13.9	52.9 ± 12.6	51.4 ± 19.7	0.971
Myer–Cotton grading						
Grade I (0–50%)	–	–	–	–	–	0.158
Grade II (50–70%)	5 (11)	2 (40)	1 (5)	1 (9)	1 (14)	
Grade III (71–99%)	40 (89)	3 (60)	21 (95)	10 (91)	6 (86)	
Grade IV (100%)	–	–	–	–	–	
Length of stenosis (mm)	20 (5–45)	20 (15–30)	20 (5–45)	25 (15–25)	25 (15–35)	0.946
Distance of stenosis to vocal folds (mm)	3 (0–30)	20 (5–30)	3 (0–15)	5 (0–15)	1 (0–3)	0.001
Total length of trachea (mm)	120 (105–135)	120 (105–130)	125 (105–135)	125 (110–130)	120 (115–130)	0.584
Aetiology						
Idiopathic	30 (67)	3 (60)	15 (68)	7 (64)	5 (72)	0.909
Acquired	8 (18)	2 (40)	4 (18)	1 (9)	1 (14)	
Autoimmune	7 (15)	–	3 (14)	3 (27)	1 (14)	
Pretreatment						
Pretreated	23 (51)	–	12 (55)	6 (55)	5 (72)	0.089
Number of treatments (range)	2 (1–9)	–	3 (1–9)	2 (1–8)	2 (1–8)	0.356
Tracheostomy at the time of surgery	3 (7)	1 (20)	–	–	2 (29)	0.024
Comorbidities						
Hypertension	10 (22)	1 (20)	4 (18)	4 (36)	1 (14)	0.649
Diabetes mellitus	3 (7)	–	1 (5)	1 (9)	1 (14)	0.755
COPD	2 (4)	1 (20)	1 (5)	–	–	0.315
Hypothyroidism	8 (18)	1 (20)	5 (23)	1 (9)	1 (14)	0.815
Hyperlipidaemia	6 (13)	2 (40)	1 (5)	2 (18)	1 (14)	0.199
Adipositas	3 (7)	1 (20)	–	1 (9)	1 (14)	0.306
Gastroesophageal reflux	3 (7)	–	2 (9)	1 (9)	–	0.779
Pulmonary embolism	1 (2)	–	1 (5)	–	–	0.802
Asthma bronchiale	1 (2)	–	–	1 (9)	–	0.388
Intracranial bleeding	1 (2)	–	1 (5)	–	–	0.802
Myocardial infarction	1 (2)	–	–	–	1 (14)	0.141
ARDS	1 (2)	1 (20)	–	–	–	0.040
Chronic renal insufficiency	1 (2)	1 (20)	–	–	–	0.040

ARDS: acute respiratory distress syndrome; COPD: chronic obstructive pulmonary disease; CTR: cricotracheal resection; SSLTR: single-stage laryngotracheal reconstruction.

### Surgical treatment

Five (11%) patients underwent standard CTR, 22 (49%) patients had additional dorsal mucosectomy followed by a dorsal mucosal flap and 11 (24%) were treated with CTR with lateral cricoplasty. A SSLTR was performed in 7 (16%) patients. These patients were characterized by a short distance of the stenosis to the glottis [median 1 mm (range 0–3); *P* = 0.001]. The overall mean operative time was 169 ± 59 min. If a SSLTR was applied, the procedural time was significantly longer 267 ± 34 min (*P* = 0.001). Overall, the total resection length was 25 mm (range 15–40) with no significant differences between groups (*P* = 0.619). At the end of surgery, 7 (16%) received a utility tracheostomy due to relevant glottic oedema, 2 of whom were in the SSLTR group (Table 2).

**Table 2:** Surgical variables and outcome in patients after cricotracheal resection and reconstruction

Variable	Total (*n* = 45)	Standard CTR (*n* = 5)	CTR with dorsal flap (*n* = 22)	CTR with lateral cricoplasty (*n* = 11)	SSLTR (*n* = 7)	*P*-value
	*n* (%)%	*n* (%)	*n* (%)	*n* (%)	*n* (%)	
Operative time (min)	169 ± 9	152 ± 49	143 ± 42	167 ± 42	267 ± 34	<0.001
Length of resection in mm (range)	25 (15–40)	30 (15–35)	25 (15–40)	25 (15–30)	25 (20–40)	0.619
Postoperative utility tracheostomy	7 (16)	2 (40)	2 (9)	1 (9)	2 (29)	0.252
Time to decannulation (days)	5 (2–9)	3 (2–4)	4.5 (3–9)	9	6.5 (5–8)	0.228
ICU stay (days)	1 (0–9)	1 (0–7)	1 (0–2)	1 (0–2)	1 (1–9)	0.143
Hospital stay (days)	6 (3–16)	4 (4–11)	6 (3–12)	6 (5–16)	8 (5–12)	0.127
Start of oral intake (days)	1 (1–11)	1 (1–5)	1 (1–4)	2 (1–2)	5 (2–11)	<0.001
Complication						
Dehiscence	–	–	–	–	–	
Wound infection	1 (2)	–	–	–	1 (14)	0.141
Soft tissue emphysema	–	–	–	–	–	
Pneumonia	–	–	–	–	–	
RLN paralysis	–	–	–	–	–	
Hospital mortality	–	–	–	–	–	
30-days mortality	–	–	–	–	–	
Follow-up						
Follow-up time (in month)	20.8 ± 13.2	11.7 ± 8.8	18.7 ± 11.4	28.1 ± 15.5	22.6 ± 13.5	0.128
Re-stenosis	–	–	–	–	–	
Lost to follow-up	2 (4)	–	1 (5)	–	1 (14)	0.529

CTR: cricotracheal resection; ICU: intensive care unit; RLN: recurrent laryngeal nerve; SSLTR: single-stage laryngotracheal reconstruction.

### Postoperative outcome

The median duration of intensive care unit stay was 1 night (range 0–9). Oral intake was started at the 1st postoperative day (range 1–11). Patients receiving a SSLTR had a significantly later start of oral intake (5 postoperative days, range 2–11; *P* < 0.001). Median hospital stay was 6 (range 3–16) days with no significant differences between groups (*P* = 0.127). There were no in-hospital or 30-day mortality. Postoperatively, a wound infection was observed in 1 (2%) patient after SSLTR, which was treated conservatively by adapting the antibiotic therapy. In patients receiving a utility tracheostomy postoperatively, decannulation was performed at a mean of the 5 postoperative days (range 2–9), without significant differences between groups (*P* = 0.228). At the 3-months follow-up bronchoscopy, complete healing without restenosis was found in all patients. The overall long-term follow-up was 20.8 ± 13.2 months with loss of follow-up in 2 (4%) patients (Table 2). All of the patients were without tracheostomy at the last follow-up. None of the patients received a reintervention for restenosis.

### Functional outcome

A complete functional follow-up could be accomplished in 42 (93%) patients. In 4 (9%) patients, postoperative vocalization took place at the vestibular fold level. Interestingly, all of these patients were in the group with SSLTR (*P* = 0.078). This voice pattern was observed already before surgery in 1 patient. In the overall cohort, there was no change in vocal fold movement (normal movement in 87% preoperatively and 89% postoperatively).

**Figure 2:**
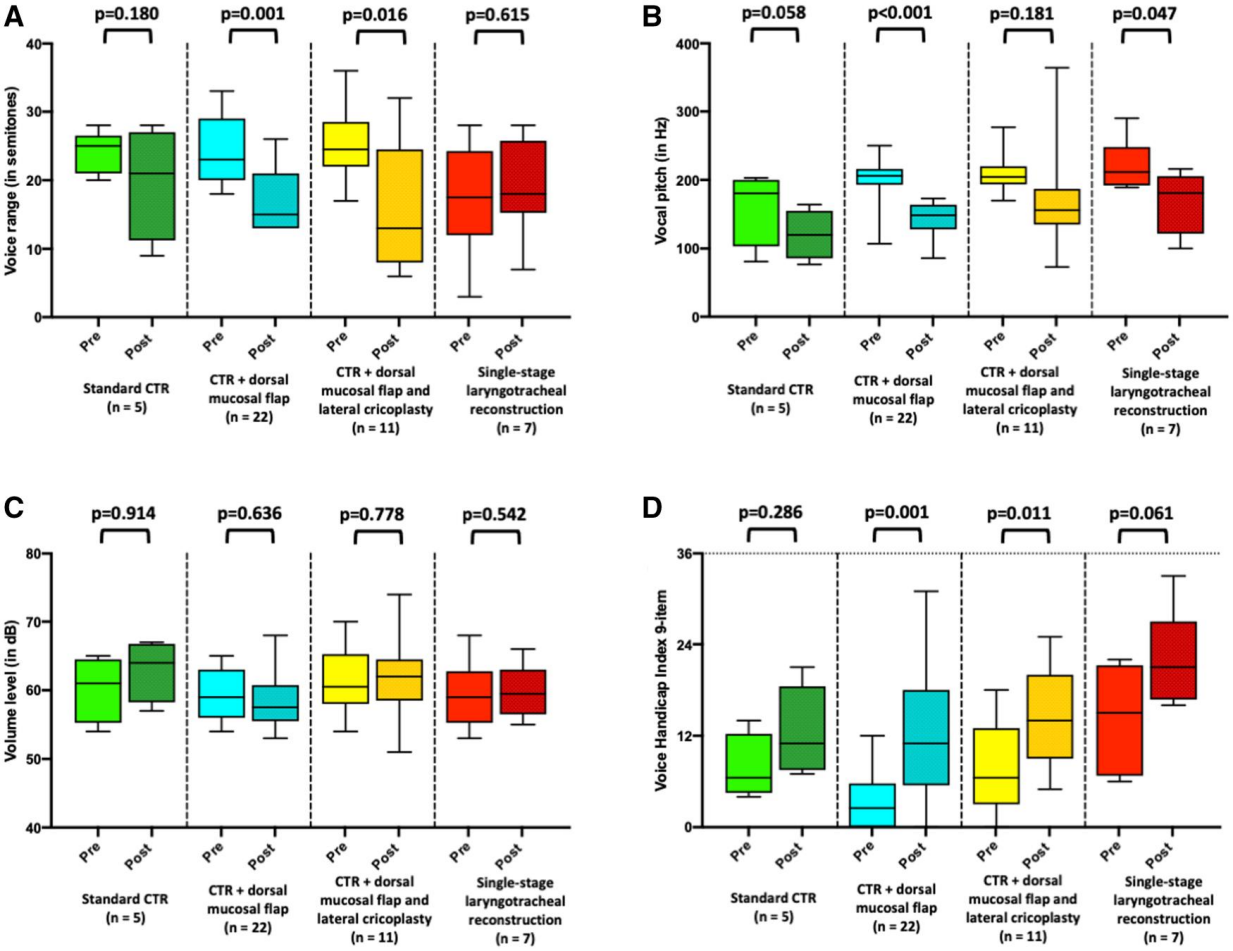
Pre- and postoperative evaluation of (**A**) voice range, (**B**) vocal pitch, (**C**) volume level, (**D**) nine-item Voice Handicap Index.

**Figure 3:**
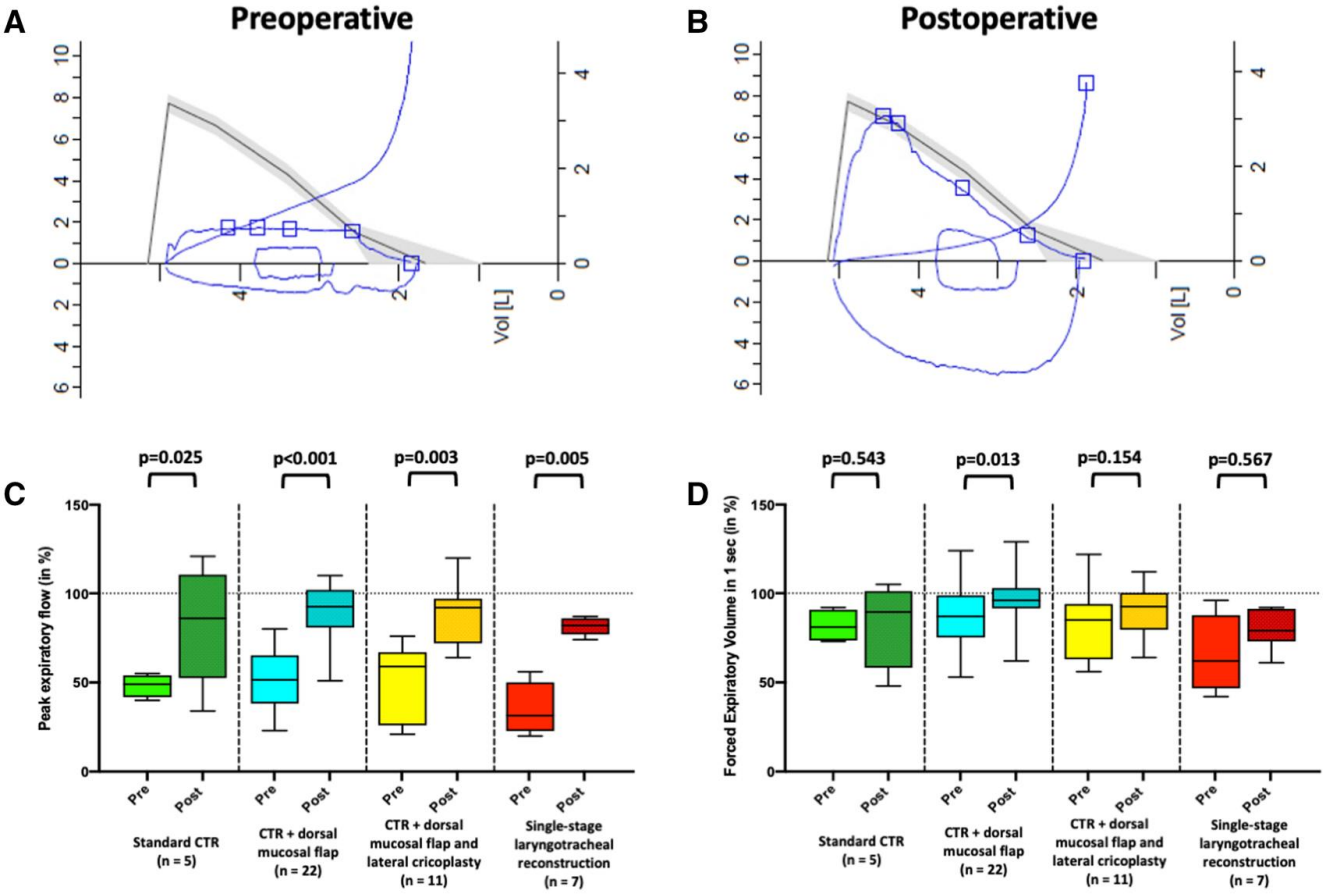
(**A**) Preoperative and (**B**) postoperative flow–volume loops after cricotracheal resection and reconstruction. Pre- and postoperative evaluation of (**C**) peak expiratory flow (in %), (**D**) forced expiratory volume in 1 s (in %).

In several (13%) patients, vocal cord mobility was already limited preoperatively due to scar bands extending to the glottis; true paresis of the recurrent laryngeal nerve was not observed in our cohort, either before or after surgery. One (14%) patient after SSLTR had postoperative findings of bilateral fixation of the vocal folds by remnant scar formations, which could not be removed during surgery. Among patients after CTR with dorsal mucosal flap (95% normal movement) and with lateral cricoplasty (91% normal movement), vocal fold mobility could actually be improved after surgery by freeing the vocal folds during surgery. Compared to the preoperative (2%) assessment, incomplete glottic closure was observed in 5 (11%) patients. One (14%) patient after SSLTR underwent vocal fold augmentation in order to improve the glottic closure and postoperative voice quality. Three months after surgery, the assessment of the Roughness–Breathiness–Hoarseness score showed significant increases in roughness (*P* < 0.001), breathiness (*P* = 0.011) and hoarseness (*P* < 0.001). The overall voice profile changed slightly from a R0B0H0 to a R1B0H1. A marked decrease of perceptive voice quality was observed in the SSLTR group with R0-1B0H1 to R2B2H2 (*P* = 0.004). The measured voice range (*P* < 0.001) decreased from 23.5 ± 5.8 to 17.8 ± 6.7 semitones in the overall study cohort (Fig. 2A). Interestingly, patients after SSLTR showed a non-significant (*P* = 0.615) change in voice range from preoperative 17.3 ± 8.6 to 19.0 ± 7.3 semitones, as the preoperative baseline measurement was already quite low. Fundamental voice pitch showed a significant (*P* < 0.001) reduction from a mean 203 (81–290) to 150 (73–364) Hz in the overall cohort (Fig. 2B). Postoperatively, no differences in fundamental voice volume were observed (60.0 ± 4.1 vs 60.2 ± 4.8 dB, *P* = 0.788) (Fig. 2C). On the nine-item VHI, there was an increasing impairment (*P* < 0.001) from 6 (0–22) to 14 (0–33) (Fig. 2D). The largest limitation was observed in the SSLTR group, which increased from an already high level of 15 (6–22) to 21 (16–33). Functional assessments of respiratory function showed a clear improvement in all groups. These improvements were accompanied by normalization of the flow–volume curves postoperatively (Fig. 3A and B). Peak expiratory flow increased significantly from 48.4 ± 17.0% preoperatively to 89.3 ± 15.4% postoperatively (*P* < 0.001) (Fig. 3C), forced expiratory volume in 1 s improved from 83.8 ± 19.4% to 90.8 ± 15.9% (*P* < 0.001) (Fig. 3D). The largest improvement in peak expiratory flow was observed in patients after SSLTR with preoperatively 34.8 ± 15.2% to 83.5 ± 3.1% postoperatively (*P* = 0.005) (Table 3).

**Table 3:** Functional outcome in patients after cricotracheal resection and reconstruction

Variable	Total (*n* = 45)		Standard CTR (*n* = 5)		CTR with dorsal flap (*n* = 22)		CTR with lateral cricoplasty (*n* = 11)		SSLTR (*n* = 7)	
	*P*-value		*P*-value		*P*-value		*P*-value		*P*-value	
Phonation										
Preoperative level of phonation		0.183		1.000		1.000		1.000		0.078
Vocal cords	44 (98)		5 (100)		22 (100)		11 (100)		6 (86)	
Vestibular cords	1 (2)		–		–		–		1 (14)	
Postoperative level of phonation										
Vocal cords	41 (91)		5 (100)		22 (100)		11 (100)		3 (43)	
Vestibular cords	4 (9)		–		–		–		4 (57)	
Preoperative vocal cord movement		0.767		1.000		0.163		0.588		0.457
Unremarkable	39 (87)		5 (100)		20 (91)		9 (82)		5 (71)	
Reduced	6 (13)		–		2 (9)		2 (18)		2 (29)	
Immobile	–		–		–		–		–	
Postoperative vocal cord movement										
Unremarkable	40 (89)		5 (100)		21 (95)		10 (91)		4 (57)	
Reduced	4 (9)		–		1 (5)		1 (9)		2 (29)	
Immobile	1 (2)		–		–		–		1 (14)	
Preoperative glottic closure		0.183		0.374		0.330		0.341		0.172
Complete	44 (96)		5 (100)		21 (95)		11 (100)		7 (100)	
Incomplete	1 (2)		–		1 (5)		–		–	
Postoperative glottic closure										
Complete	40 (89)		4 (80)		21 (95)		10 (91)		5 (71)	
Incomplete	5 (11)		1 (20)		1 (5)		1 (9)		2 (29)	
RBH-score										
Preoperative roughness		<0.001		0.391		<0.001		0.031		0.015
Grade 0	27 (60)		3 (60)		14 (63)		7 (64)		3 (43)	
Grade 1	15 (34)		2 (40)		7 (32)		3 (27)		3 (43)	
Grade 2	1 (2)		–		–		1 (9)		–	
Grade 3	1 (2)		–		–		–		1 (14)	
N/A	1 (2)		–		1 (5)		–		–	
Postoperative roughness										
Grade 0	7 (15)		2 (40)		2 (9)		3 (27)		–	
Grade 1	23 (51)		2 (40)		16 (73)		4 (37)		1 (14)	
Grade 2	9 (20)		–		2 (9)		3 (27)		4 (57)	
Grade 3	3 (7)		–		–		1 (9)		2 (29)	
N/A	3 (7)		1 (20)		2 (9)		–		–	
Preoperative breathiness		0.011		0.391		0.135		0.341		0.078
Grade 0	33 (74)		4 (80)		16 (73)		9 (82)		4 (57)	
Grade 1	10 (22)		1 (20)		5 (22)		2 (18)		2 (29)	
Grade 2	–		–		–		–		–	
Grade 3	1 (2)		–		–		–		1 (14)	
N/A	1 (2)		–		1 (5)		–		–	
Postoperative Breathiness										
Grade 0	25 (56)		3 (60)		13 (59)		8 (73)		1 (14)	
Grade 1	9 (20)		1 (20)		5 (23)		2 (18)		1 (14)	
Grade 2	7 (15)		–		2 (9)		–		5 (72)	
Grade 3	1 (2)		–		–		1 (9)		–	
N/A	3 (7)		1 (20)		2 (9)		–		–	
Preoperative hoarseness		<0.001		0.182		<0.001		0.031		0.018
Grade 0	25 (56)		3 (60)		13 (59)		7 (64)		2 (29)	
Grade 1	17 (38)		2 (40)		8 (36)		3 (27)		4 (57)	
Grade 2	1 (2)		–		–		1 (9)		–	
Grade 3	1 (2)		–		–		–		1 (14)	
N/A	1 (2)		–		1 (5)		–		–	
Postoperative hoarseness										
Grade 0	6 (13)		1 (20)		2 (9)		3 (27)		–	
Grade 1	24 (53)		3 (60)		16 (73)		4 (37)		1 (14)	
Grade 2	9 (20)		–		2 (9)		3 (27)		4 (57)	
Grade 3	3 (7)		–		–		1 (9)		2 (29)	
N/A	3 (7)		1 (20)		2 (9)		–		–	
Preoperative voice range (semitones)	23.5 ± 5.8	<0.001	24.0 ± 3.1	0.180	24.5 ± 4.5	0.001	25.0 ± 5.4	0.016	17.3 ± 8.6	0.615
Postoperative voice range (semitones)	17.8 ± 6.7		19.7 ± 8.3		17.9 ± 5.0		16.4 ± 9.4		19.0 ± 7.3	
Preoperative vocal pitch (Hz)	203 (81–290)	<0.001	191 (136–215)	0.200	203 (81–250)	<0.001	206 (170–277)	0.018	211 (189–290)	0.132
Postoperative vocal pitch (Hz)	150 (73–364)		128 (112–142)		149 (77–173)		156 (73–364)		181 (100–216)	
Preoperative volume level (dB)	60.0 ± 4.1	0.788	59.5 ± 4.0	0.914	59.5 ± 3.5	0.636	62.2 ± 4.0	0.778	59.3 ± 5.1	0.542
Postoperative volume level (dB)	60.2 ± 4.8		59.2 ± 2.6		59.1 ± 4.4		61.6 ± 6.2		59.8 ± 3.9	
Preoperative 9-Voice-Handicap-Index	6 (0–22)	<0.001	7 (1–14)	0.127	4 (0–18)	<0.001	7 (0–18)	0.179	15 (6–22)	0.132
Postoperative 9-Voice Handicap Index	14 (0–33)		7 (11–21)		11 (0–31)		14 (5–25)		21 (16–33)	
Preoperative phonation time (sec)	17 (8–38)	0.183	8 (8–20)	0.999	17 (9–32)	0.319	17 (9–38)	0.303	18 (10–31)	0.906
Postoperative phonation time (sec)	18 (7–35)		7 (12–23)		18 (8–35)		21 (10–33)		19 (10–30)	
Swallowing										
Preoperative swallowing		0.323		1.000		1.000		0.341		1.000
Unremarkable	44 (98)		5 (100)		21 (95)		11 (100)		7 (100)	
Aspiration	–		–		–		–		–	
Penetration	–		–		–		–		–	
N/A	1 (2)		–		1 (5)		–		–	
Postoperative swallowing										
Unremarkable	42 (93)		4 (80)		20 (91)		11 (100)		7 (100)	
Aspiration	–		–		–		–		–	
Penetration	–		–		–		–		–	
N/A	3 (7)		1 (20)		2 (9)		–		–	
Preoperative dysphagia self rating 1–7	1 (1–3)	0.168	1 (1–3)	0.999	1 (1–2)	0.482	1 (1–2)	0.201	1 (1–2)	0.999
Postoperative dysphagia self rating 1–7	1 (1–4)		1 (1–2)		1 (1–3)		2 (1–4)		1 (1–2)	
Spirometry										0
Preoperative PEF (%)	48.4 ± 17.0	<0.001	48.2 ± 6.3	0.025	50.3 ± 17.3	<0.001	51.7 ± 20.0	0.003	34.8 ± 15.2	0.005
Postoperative PEF (%)	89.3 ± 15.4		94.5 ± 21.3		88.8 ± 16.0		90.5 ± 16.3		83.5 ± 3.1	
Preoperative FEV1 (%)	83.8 ± 19.4	<0.001	84.2 ± 8.0	0.543	88.1 ± 19.1	0.013	83.1 ± 19.8	0.154	65.5 ± 22.4	0.567
Postoperative FEV1 (%)	90.8 ± 15.9		87.2 ± 22.7		97.0 ± 14.3		90.7 ± 14.5		79.8 ± 11.3	

CTR: cricotracheal resection; FEV1: forced expiratory volume in 1 s; PEF: peak expiratory flow; RBH: Roughness–Breathiness–Hoarseness; N/A: not assessed.

**Figure 4:**
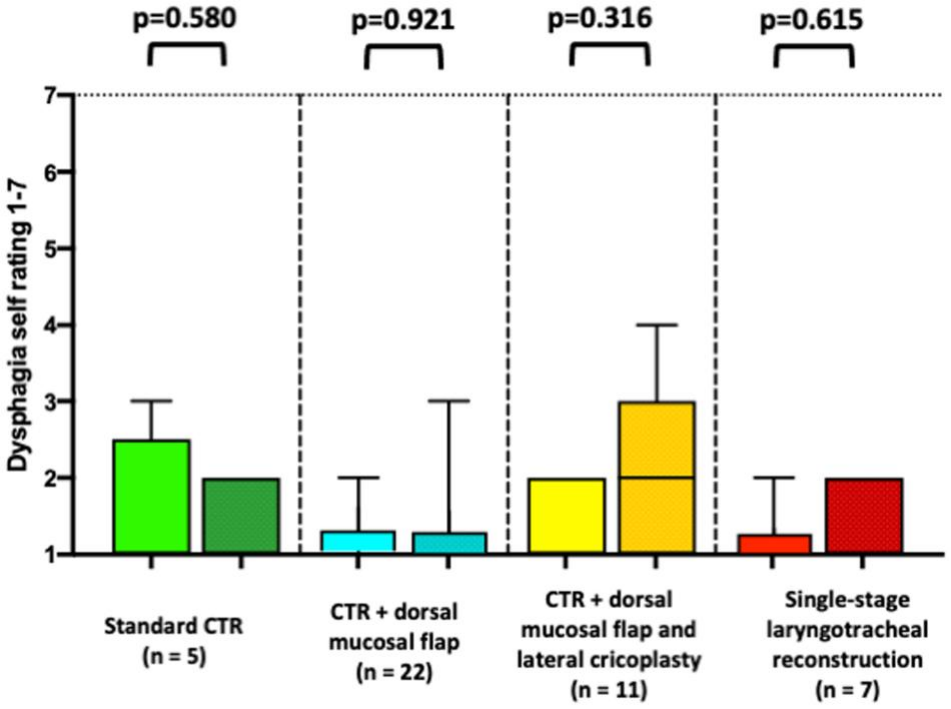
Preoperative and postoperative dysphagia self-rating (1–7).

At the postoperative follow-up visit, all patients had sufficient deglutition and full oral intake. In fibreoptic endoscopic evaluation of swallowing, there was no indication of aspiration or penetration in any patient. There were no significant differences in patient self-rating for dysphagia from 1 to 7 in all groups [1 (1–3) vs 1 (1–4), *P* = 0.168] (Fig. 4).

## DISCUSSION

In this work, we could demonstrate the impact of various types of laryngotracheal procedures on the postoperative functional outcome in patients with benign, subglottic stenosis. We focused particularly on the 3 laryngeal functions, namely voice, swallowing and respiration. This is to the best of our knowledge the so far largest in-depth functional analysis from a single high-volume centre of airway surgery.

In experienced hands, laryngotracheal procedures can be carried out with extremely low peri-operative morbidity. The prevailing aim to restore the respiratory capacity and avoiding permanent tracheostomy could be achieved in all of the patients in the present study. At a median follow-up of 20.8 ± 13.2 months, none of our patients experienced restenosis. This underlines the value of open-surgical procedures as a definite treatment option in these patients.

Previous studies described the impact of subglottic resection on the postoperative voice outcome. In a study of 16 patients after CTR by Houlton *et al.*, a decrease in fundamental vocal pitch from 194.9 to 152.7 Hz (*P* = 0.047) was observed. These results are consistent with our data, which show a lowering of the fundamental vocal pitch by ∼40–50 Hz [16–18]. In addition, there was a lowering of dynamic voice amplitude by ∼5–8 semitones. Similar results were reported by Smith *et al*. in a group of 14 women after CTR. This study showed a reduction in voice range of ∼5.9 semitones from 21.5 to 15.6 (*P* = 0.05) [3]. Interestingly, in our study, a nonsignificant increase in vocal range by 2 semitones was observed in patients after SSLTR. Another key finding was that fundamental vocal volume was fully preserved throughout all groups.

Our data suggest that increasing surgical complexity and invasiveness is paralleled by an increasing deterioration of voice quality. This is consistent with the findings of Pullens *et al*. who identified glottic involvement as a key predictor of poor functional outcome [19]. Exceptions in our study are patients with glotto-subglottic stenosis with an already low baseline voice quality.

Interestingly, the mobility of the vocal folds could be even restored in some patients with cicatricial strands to the vocal folds. However, the concomitant removal of the cricoid arch leads to a decreased tension in the vocal fold and therefore to a lower fundamental frequency. This illustrates the interplay and peculiarities of several anatomical structures in the larynx affecting the overall voice quality.

The findings in our work has also implications on patient education and pre-operative informed consent. Patients receiving CTRs or more complex procedures have to be informed that, at least a modest decline in vocal pitch and voice range have to be expected. This might be relevant especially in patients working in positions where a loud and clear voice is necessary [1].

Interestingly, the permanent restoration of the airway outweighs these moderate impairments of the voice capacity. A significant improvement in peak expiratory flow from 48.4% to 89.3% (*P* < 0.001) was observed in our study over all groups. Again, the highest increase was seen in patients after SSLTR, with preoperative 34.8% to postoperative 83.5%. The subjective gain of quality of life after surgery exceeds the quality of life after endoscopic procedures. Quality of life based on the Clinical Chronic Obstructive Pulmonary Disease Questionnaire (CCQ) was superior after CTR with 0.75 vs 1.8 after endoscopic treatment in a large multicentre study of 810 patients, despite limitation in the VHI-10 with 13 vs 6 points [1].

It is still an ongoing discussion if and when surgery should be offered to patients with laryngotracheal stenosis [20, 21]. Traditionally, at least 1 endoscopic treatment attempt was suggested before referring a patient for an open repair. But the rate of stenosis recurrence is high. In a systematic review, Lavrysen *et al*. reported a rate of restenosis after endoscopic interventions of 68% on average [22]. However, in our experience, any trauma to the subglottic region adds further complexity to a possible surgical repair. Treatment-naive patients diagnosed with idiopathic subglottic stenosis and without any re-canalization attempts present typically with a short-segment stenosis located at the height of the 1st tracheal rings and involve only the lower border of the cricoid. In these patients, almost the entire cricoid can be preserved with hardly any impact on the laryngeal skeleton and thus voice quality. Therefore, the traditional management of at least 1 endoscopic treatment attempt should be questioned. The rationale that surgery can always be offered after failure of the less-invasive endoscopic treatment is obviously right, but on the other hand the associated added complexity of surgery should not be neglected. A paper by Nouraei *et al*. already demonstrated the progression of proximal stenosis after repetitive endoscopic treatments, which can even result in vocal fold fixation [23]. In our opinion, upfront surgery at an experienced institution should be offered also to patients with treatment-naive, subglottic stenosis.

### Limitations

This study is subject to several limitations. First of all, the retrospective design of this study might limit the possible conclusions. However, functional measurements were acquired in a standardized manner and uniformly applied in all patients. Despite the relatively high annual caseload at our centre, the overall number of patients in context of statistical power is still limited. To overcome this limitation, multicentric studies with well-defined standardized outcome parameters are required and in preparation. For this purpose, a multicentre AIR database has already been established in cooperation with the European Society of Thoracic Surgeons (ESTS) and 1st findings will be published soon [24].

## CONCLUSION

In conclusion, even high-grade and complex airway stenosis can be treated with excellent surgical and good functional outcome. However, functional outcome is inversely associated with the complexity of the surgical procedure. Notably, among complex procedures, a majority of patients were pretreated (CTR with lateral cricoplasty 55%; SSLTR 72%), some of whom had up to 8 treatments before final surgical therapy. Thus, referral to possible surgery should be considered early in the treatment of patients with laryngotracheal stenosis.

## Supplementary Material

ezae171_Supplementary_Data

## Data Availability

The data underlying this article are available in the article and in its online supplementary material.

## ABBREVIATIONS

CCQ: Clinical Chronic Obstructive Pulmonary Disease Questionnaire
CTR: cricotracheal resection
ESTS: European Society of Thoracic Surgeons
SSLTR: single-stage laryngotracheal reconstruction
VHI: Voice Handicap Index
