# Supplementary material for: In-depth analysis of pre- and postoperative functional outcome parameters in patients receiving laryngotracheal surgery
Source: Eur J Cardiothorac Surg. 2024 Apr 19;65(5):ezae171. doi: 10.1093/ejcts/ezae171 (PMC11078893; doi:10.1093/ejcts/ezae171)
Supplement: ezae171_Supplementary_Data [file ezae171_supplementary_data.zip › supplementary material.docx]

**Supplementary material**

## **Supplementary table**

| **Variable** | **Anderson-Darling test (p-value)** |
| --- | --- |
| Median age of surgery | 0.829 |
| Length of stenosis | 0.003 |
| Distance of stenosis to vocal folds | <0.001 |
| Total length of trachea | 0.014 |
| Operative time | 0.067 |
| Length of resection | 0.004 |
| ICU stay | <0.001 |
| Hospital stay | <0.001 |
| Start of oral intake | <0.001 |
| Follow-up in month | 0.104 |
| Voice range | 0.190 |
| Vocal pitch | 0.001 |
| Volume level | 0.606 |
| 9-Voice-Handicap-Index | 0.002 |
| Phonation time | 0.019 |
| Dysphagia self rating | <0.001 |
| PEF | 0.191 |
| FEV1 | 0.601 |

## **Legend**

Supplementary table: Testing of normal distribution (Anderson-Darling test)

Supplementary figure: 3-month follow-up bronchoscopy after single-stage laryngotracheal reconstruction.
